# Fast Number Theoretic Transform for Ring-LWE on 8-bit AVR Embedded Processor

**DOI:** 10.3390/s20072039

**Published:** 2020-04-05

**Authors:** Hwajeong Seo, Hyeokdong Kwon, Yongbeen Kwon, Kyungho Kim, Seungju Choi, Hyunjun Kim, Kyoungbae Jang

**Affiliations:** Hansung University, IT Engineering, 116 Samseong-Yoro-16-Gil Seongbuk-gu, Seoul 136-792, Korea; hyeok@hansung.ac.kr (H.K.); soono99@hansung.ac.kr (Y.K.); pgmkkh@hansung.ac.kr (K.K.); bookingstore3@hansung.ac.kr (S.C.); amdjd0704@hansung.ac.kr (H.K.); starj1234@hansung.ac.kr (K.J.)

**Keywords:** ring learning with errors, software implementation, public key encryption, 8-bit AVR microcontroller, AES block cipher, random sampling, number theoretic transform, timing attack

## Abstract

In this paper, we optimized Number Theoretic Transform (NTT) and random sampling operations on low-end 8-bit AVR microcontrollers. We focused on the optimized modular multiplication with secure countermeasure (i.e., constant timing), which ensures high performance and prevents timing attack and simple power analysis. In particular, we presented combined Look-Up Table (LUT)-based fast reduction techniques in a regular fashion. This novel approach only requires two times of LUT access to perform the whole modular reduction routine. The implementation is carefully written in assembly language, which reduces the number of memory access and function call routines. With LUT-based optimization techniques, proposed NTT implementations outperform the previous best results by 9.0% and 14.6% for 128-bit security level and 256-bit security level, respectively. Furthermore, we adopted the most optimized AES software implementation to improve the performance of pseudo random number generation for random sampling operation. The encryption of AES-256 counter (CTR) mode used for random number generator requires only 3184 clock cycles for 128-bit data input, which is 9.5% faster than previous state-of-art results. Finally, proposed methods are applied to the whole process of Ring-LWE key scheduling and encryption operations, which require only 524,211 and 659,603 clock cycles for 128-bit security level, respectively. For the key generation of 256-bit security level, 1,325,171 and 1,775,475 clock cycles are required for H/W and S/W AES-based implementations, respectively. For the encryption of 256-bit security level, 1,430,601 and 2,042,474 clock cycles are required for H/W and S/W AES-based implementations, respectively.

## 1. Introduction

The hard problem of traditional Public Key Cryptography (PKC) algorithms, such as RSA and Elliptic Curve Cryptography (ECC), rely on Integer Factorization (IF) and Discrete Logarithm Problem (DLP), which have been believed to be secure and robust against any classical attacks on traditional computer settings until now. For this reason, these traditional PKC protocols have been widely deployed, such as SSL, TLS, and HTTPS. Since the PKC protocols are more expensive than symmetric key cryptography, many previous works focused on high-performance and compact implementations of PKC algorithms (e.g., RSA and ECC) on various platforms [1,2,3,4,5,6,7,8,9,10,11,12,13,14,15,16,17,18,19].

However, such a problem that is believed to be hard can be easily broken by using Shor’s algorithm in a polynomial time when large-scale quantum computers and practical quantum algorithms are developed [20]. Recently, global leading IT companies, such as Google and IBM, introduced new quantum computer architectures, which shows that quantum attack on the quantum computer would be feasible in the near future. In order to avoid these potential quantum threats, many quantum-resistant cryptography algorithms have been studied, which includes code-based cryptography, lattice-based cryptography, multivariate-based cryptography, hash-based cryptography, and Isogeny-based cryptography. However, code-based cryptography and multivariate-based cryptography the have long key problem. Hash-based cryptography has long signature problem. Isogeny-based cryptography has long execution timing. Among them, the lattice-based cryptography is considered as one of the most promising candidates for post-quantum cryptography due to its reasonably small key size, small cipher text size, and short execution timing. The lattice-based cryptography is built based on worst-case computational assumptions, such as Shortest Vector Problem (SVP) and Closest Vector Problem (CVP) in lattices, that would still remain a hard problem even for well-known quantum algorithms over quantum computers.

Furthermore, future computing environments, such as Internet of Things (IoT), would be widely deployed and used in near future for applications. In particular, low-end IoT devices are deployed in remote areas and need to handle huge sensor data. The collected data is processed and used for useful applications since the data may include sensitive information. For this reason, secure cryptographic algorithms should be implemented on low-end IoT devices to ensure integrity and confidentiality of data. However, low-end IoT devices are usually very resource-constrained, in terms of computing power, energy, ROM, and RAM. These hard conditions are a challenge to implement the cryptography algorithm in an efficient manner on low-end devices. In this paper, we present the most optimal implementation of Number Theoretic Transform (NTT) computation and random sampling operation for lattice-based cryptography on target platforms. The implementation also ensures the constant timing through the regular pattern of program, which is secure against timing attack and simple power analysis. Furthermore, we adopted the most optimized AES software implementation to improve the performance of pseudo random number generation. The encryption of AES-256 counter mode requires 3,184 clock cycles, which is 9.5% faster than previous state-of-art results. This approach efficiently improves the performance of random sampler.

The introduction of Learning with Errors (LWE) problem and its Ring-based scheme (Ring-LWE) provided efficient ways to construct the lattice-based public key cryptography for post-quantum cryptography [21,22]. There are many works to improve lattice-based cryptography on low-end microcontrollers. In particular, software implementations of Ring-LWE-based public-key encryption or digital signature schemes mainly focused on the improvements of execution timing and memory requirements. In DAC’14, Oder et al. presented an efficient implementation of Bimodal Lattice Signature Scheme (BLISS) on a 32-bit ARM Cortex-M4F microcontroller [23]. This is the first optimized implementation of lattice-based signature scheme on 32-bit low-end ARM processor. In DATE’15, De Clercq et al. implemented Ring-LWE encryption scheme on the identical 32-bit ARM Cortex-M4F microcontroller [24]. They utilized 32-bit registers to maintain two 13~14-bit coefficients at once rather than separated way. The parallel approach improved the performance and register utilization, significantly. Boorghany et al. implemented lattice-based cryptography schemes on the low-end 8-bit AVR microcontroller for the first time [25,26]. The authors evaluated several lattice-based authentication protocols on both 8-bit AVR and 32-bit ARM microcontrollers. In particular, Fast Fourier Transform (FFT) transform and Gaussian sampler function are implemented with optimized multiplication and sampling operations. In LATINCRYPT’15, Pöppelmann et al. introduced implementations of Ring-LWE encryption and BLISS on low-end 8-bit AVR ATxmega128 microcontrollers [27]. In CHES’15, Liu et al. optimized implementations of Ring-LWE encryption by presenting efficient modular multiplication, NTT computation, and refined memory access schemes to achieve high performance and low memory consumption for 8-bit AVR microcontrollers [28]. They presented two implementations of Ring-LWE encryption scheme for short-term (128-bit) and long-term (256-bit) security levels on low-end 8-bit AVR microcontrollers. They also presented the optimized sampling technique using AES accelerator for high speed random number generation. In [29], Liu et al. presented the first secure Ring-LWE encryption and BLISS signature implementations against timing attacks and simple power analysis. NTT and sampling operations are implemented in constant timing to prevent timing attack and simple power analysis. Particularly, the modular reduction is performed in Montgomery reduction to reduce computation complexity, significantly. The sampling ensures constant timing or random shuffling. In ICISC’17, Seo et al. proposed secure and efficient Ring-LWE implementations by using LUT-based modular reduction technique and random shuffling [30]. This is the first approach to utilize the LUT for reduction. Recently, in WISA’19, Seo et al. presented novel LUT-based modular reduction method [31]. Unlike the previous work in [30], this approach only requires two times of LUT access to perform the modular reduction by modifying the input value.

### 1.1. Extended Version of WISA’19

In this paper, we extended our previous work published in WISA’19 [31]. In the previous work, only Ring-LWE implementations for long term security level (256-bit) were investigated. The work mainly optimized the modular reduction on 12,289 prime. In this extended work, we further investigated ring-LWE implementations for short term security level (128-bit). This implementation focused on optimization of modular multiplication on 7681 prime. Furthermore, we fully optimized the modular multiplication by using assembly language. In addition, we provided new random number generator using the fastest AES software implementation, namely FACE-LIGHT [32]. This approach improves the performance of AES-based random number generator for random sampling.

### 1.2. Contributions

The secure and compact Ring-LWE encryption scheme on the low-end 8-bit AVR microcontroller is proposed. The implementation is optimized with efficient techniques and the countermeasure against simple power analysis and timing attack is given. The Number Theoretic Transform (NTT)-based polynomial multiplication is optimized with the Look-Up Table (LUT)-based modular multiplication approach. This new approach performs the modular reduction with two LUT accesses. Furthermore, we adopted the most optimized AES software implementation, namely FACE-LIGHT, to improve the performance of Pseudo Random Number Generation (PRNG). This is based on knowledge that repetition of some data in counter mode. The PRNG is used to improve the performance of random sampling. With above optimized techniques, Ring-LWE implementations on 8-bit AVR microcontroller are highly accelerated.

The key scheduling requires 524 K, 1325 K, and 1775 K for 128-bit security level with hardware AES-128, 256-bit security level with hardware AES-128, and 256-bit security level with software AES-256, respectively. The encryption requires 659 K, 1430 K, and 2042 K for 128-bit security level with hardware AES-128, 256-bit security level with hardware AES-128, and 256-bit security level with software AES-256, respectively.

The paper consists of 5 sections. In Section 2, the background of Ring-LWE, NTT, and previous works are covered. In Section 3, optimized implementations of modular reduction for NTT and fast AES encryption of random sampling on low-end 8-bit AVR microcontrollers are introduced. For the high performance random sampling, the most optimized AES software implementation (FACE-LIGHT) is used. In Section 4, the performance of proposed implementation is given. Furthermore, the result is compared with state-of-the-art implementations. Finally, the paper is concluded in Section 5.

## 2. Introduction

The hard problem of traditional Public Key Cryptography (PKC) algorithms, such as RSA and Elliptic Curve Cryptography (ECC), rely on Integer Factorization (IF) and Discrete Logarithm Problem (DLP), which have been believed to be secure and robust against any classical attacks on traditional computer settings until now. For this reason, these traditional PKC protocols have been widely deployed, such as SSL, TLS, and HTTPS. Since the PKC protocols are more expensive than symmetric key cryptography, many previous works focused on high-performance and compact implementations of PKC algorithms (e.g., RSA and ECC) on various platforms [1,2,3,4,5,6,7,8,9,10,11,12,13,14,15,16,17,18,19].

However, such problems that are believed to be hard can be easily broken by using Shor’s algorithm in a polynomial time when large-scale quantum computers and practical quantum algorithms are developed [20]. Recently, global leading IT companies, such as Google and IBM, introduced new quantum computer architectures, which shows that quantum attack on the quantum computer would be feasible in near future. In order to avoid these potential quantum threats, many quantum-resistant cryptography algorithms have been studied, which includes code-based cryptography, lattice-based cryptography, multivariate-based cryptography, hash-based cryptography, and Isogeny-based cryptography. However, code-based cryptography and multivariate-based cryptography have long key problem. Hash-based cryptography has long signature problem. Isogeny-based cryptography has long execution timing. Among them, the lattice-based cryptography is considered as one of the most promising candidates for post-quantum cryptography due to its reasonably small key size, small cipher text size, and short execution timing. The lattice-based cryptography is built based on worst-case computational assumptions, such as Shortest Vector Problem (SVP) and Closest Vector Problem (CVP) in lattices, that would still remain hard problem even for well-known quantum algorithms over quantum computers.

Furthermore, future computing environments, such as Internet of Things (IoT), would be widely deployed and used in near future for applications. In particular, low-end IoT devices are deployed in remote areas and need to handle huge sensor data. The collected data is processed and used for useful applications since the data may include sensitive information. For this reason, secure cryptographic algorithms should be implemented on low-end IoT devices to ensure integrity and confidentiality of data. However, low-end IoT devices are usually very resource-constrained, in terms of computing power, energy, ROM, and RAM. These hard conditions are a challenge to implement the cryptography algorithm in an efficient manner on low-end devices. In this paper, we present the optimal implementation of NTT computation and random sampling operation for lattice-based cryptography on target platforms. The implementation also ensures the constant timing through the regular pattern of program, which is secure against timing attack and simple power analysis. Furthermore, we adopted the most optimized AES software implementation to improve the performance of pseudo random number generation. The encryption of AES-256 counter mode requires 3184 clock cycles, which is 9.5% faster than previous state-of-art results. This approach efficiently improves the performance of random sampler.

The introduction of Learning with Errors (LWE) problem and its Ring-based scheme (Ring-LWE) provided efficient ways to construct the lattice-based public key cryptography for post-quantum cryptography [21,22]. There are many works to improve lattice-based cryptography on low-end microcontrollers. In particular, software implementations of Ring-LWE-based public-key encryption or digital signature schemes mainly focused on the improvements of execution timing and memory requirements. In DAC’14, Oder et al. presented an efficient implementation of Bimodal Lattice Signature Scheme (BLISS) on a 32-bit ARM Cortex-M4F microcontroller [23]. This is the first optimized implementation of lattice-based signature scheme on 32-bit low-end ARM processor. In DATE’15, De Clercq et al. implemented Ring-LWE encryption scheme on the identical 32-bit ARM Cortex-M4F microcontroller [24]. They utilized 32-bit registers to maintain two 13~14-bit coefficients at once rather than separated way. The parallel approach improved the performance and register utilization, significantly. Boorghany et al. implemented lattice-based cryptography schemes on the low-end 8-bit AVR microcontroller for the first time [25,26]. The authors evaluated several lattice-based authentication protocols on both 8-bit AVR and 32-bit ARM microcontrollers. In particular, Fast Fourier Transform (FFT) transform and Gaussian sampler function are implemented with optimized multiplication and sampling operations. In LATINCRYPT’15, Pöppelmann et al. introduced implementations of Ring-LWE encryption and BLISS on low-end 8-bit AVR ATxmega128 microcontrollers [27]. In CHES’15, Liu et al. optimized implementations of Ring-LWE encryption by presenting efficient modular multiplication, NTT computation, and refined memory access schemes to achieve high performance and low memory consumption for 8-bit AVR microcontrollers [28]. They presented two implementations of Ring-LWE encryption scheme for short-term (128-bit) and long-term (256-bit) security levels on low-end 8-bit AVR microcontrollers. They also presented the optimized sampling technique using AES accelerator for high speed random number generation. In [29], Liu et al. presented the first secure Ring-LWE encryption and BLISS signature implementations against timing attacks and simple power analysis. NTT and sampling operations are implemented in constant timing to prevent timing attack and simple power analysis. Particularly, the modular reduction is performed in Montgomery reduction to reduce computation complexity, significantly. The sampling ensures constant timing or random shuffling. In ICISC’17, Seo et al. proposed secure and efficient Ring-LWE implementations by using LUT-based modular reduction technique and random shuffling [30]. This is the first approach to utilize the LUT for reduction. Recently, in WISA’19, Seo et al. presented novel LUT-based modular reduction method [31]. Unlike the previous work in [30], this approach only requires two times of LUT access to perform the modular reduction by modifying the input value.

### 2.1. Extended Version of WISA’19

In this paper, we extended our previous work published in WISA’19 [31]. In the previous work, only Ring-LWE implementations for long term security level (256-bit) were investigated. The work mainly optimized the modular reduction on 12,289 prime. In this extended work, we further investigated ring-LWE implementations for short term security level (128-bit). This implementation focused on optimization of modular multiplication on 7681 prime. Furthermore, we fully optimized the modular multiplication by using assembly language. In addition, we provided new random number generator using the fastest AES software implementation, namely FACE-LIGHT [32]. This approach improves the performance of AES-based random number generator for random sampling.

### 2.2. Contributions

The secure and compact Ring-LWE encryption scheme on the low-end 8-bit AVR microcontroller is proposed. The implementation is optimized with efficient techniques and the countermeasure against simple power analysis and timing attack is given. Number Theoretic Transform (NTT)-based polynomial multiplication is optimized with Look-Up Table (LUT)-based modular multiplication approach. This new approach performs the modular reduction with two LUT accesses. Furthermore, we adopted the most optimized AES software implementation, namely FACE-LIGHT, to improve the performance of Pseudo Random Number Generation (PRNG). This is based on knowledge that repetition of some data in counter mode. The PRNG is used to improve the performance of random sampling. With above optimized techniques, Ring-LWE implementations on 8-bit AVR microcontroller are highly accelerated.

The key scheduling requires 524 K, 1325 K, and 1775 K for 128-bit security level with hardware AES-128, 256-bit security level with hardware AES-128, and 256-bit security level with software AES-256, respectively. The encryption requires 659 K, 1430 K, and 2042 K for 128-bit security level with hardware AES-128, 256-bit security level with hardware AES-128, and 256-bit security level with software AES-256, respectively.

The paper consists of 5 sections. In Section 2, the background of Ring-LWE, NTT, and previous works are covered. In Section 3, optimized implementations of modular reduction for NTT and fast AES encryption of random sampling on low-end 8-bit AVR microcontrollers are introduced. For the high performance random sampling, the most optimized AES software implementation (FACE-LIGHT) is used. In Section 4, the performance of proposed implementation is given. Furthermore, the result is compared with state-of-the-art implementations. Finally, the paper is concluded in Section 5.

## 3. Proposed Methods

### 3.1. Look-Up Table-Based Fast Reduction

The NTT computation uses the majority of the execution time on the modular multiplication operation since it is performed in the innermost *k*-loop of the NTT computation (Step 6~9 of Algorithm 1). For this reason, optimized implementations of these steps are main concern for high speed implementation on low-end 8-bit AVR microcontrollers.

The Ring-LWE scheme utilizes 13~14-bit coefficients. The efficient implementation of operations over these bits is important. Firstly, the 16-bit wise multiplication can be efficiently performed by using the Move-and-Add method presented in [28]. For the modular reduction, many optimized techniques have been considered since the modular reduction consumes majority of execution time in modular multiplication of Ring-LWE coefficients.
**Algorithm 1** Iterative Number Theoretic Transform**Require:** A polynomial $a\left(x\right)\in Z_{q}\left[x\right]$ of degree n−1 and *n*-th primitive $\omega \in Z_{q}$ of unity**Ensure:** Polynomial $a\left(x\right)=NTT\left(a\right)\in Z_{q}\left[x\right]$1: a=BitReverse(a)2: **for**
*i* from 2 by i=2i to *n*
**do**3:  $\omega_{i}=\omega_{n}^{n/i}$, ω=14:  **for**
*j* from 0 by 1 to i/2−1
**do**5:   **for**
*k* from 0 by *i* to n−1
**do**6:    U=a[k+j]7:    V=ω·a[k+j+i/2]8:    a[k+j]=U+V9:    a[k+j+i/2]=U−V10:   $\omega =\omega \cdot \omega_{i}$11: **return**
*a*

### 3.2. Number Theoretic Transform

In this paper, we optimized the modular reduction for the high-speed implementation of NTT computation. We chose q=7681 and q=12,289 primes (i.e., 0x1E01 and 0x3001 in hexadecimal representation) for the target parameters, which are widely used in previous works [28,29,30].

The modular reduction can be implemented using the bit-shift and add technique (i.e., SAMS2) or Montgomery reduction covered in previous works [28,29]. These methods can be accelerated further by using the optimized Look-Up Table (LUT) access-based fast reduction technique for performing mod 7681 and mod 12,289 operations in ICISC’17 [30]. The main idea of the LUT-based approach is to first reduce the result by using 8-bit wise pre-computed reduced results. Afterward, the tiny fast reduction steps are performed on short coefficients. The results are kept in the incomplete representation in order to optimize the number of subtraction operations in the reduction step. This approach is a well-known lazy reduction technique and ensures that last step will go through the complete reduction. In this paper, we further optimized the LUT-based approach by using novel combined (or well aligned) LUT techniques.

**Figure 1:**
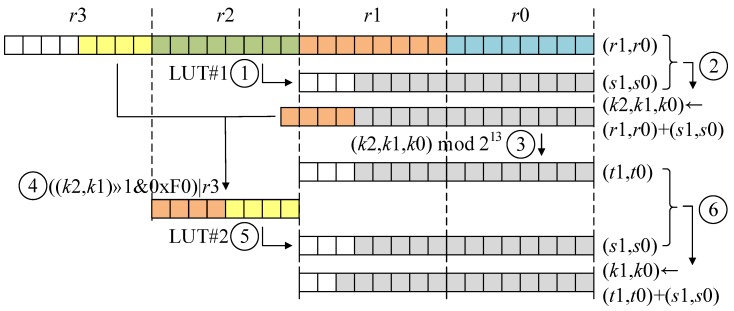
Look-Up Table (LUT)-based Fast Reduction for q=7681, ①: LUT access; ②: addition; ③: modulo; ④: concatenation; ⑤: LUT access; ⑥: addition.

When the target prime modulus is q=7681, the operand is located within range (0~0x3FFF). The intermediate result of partial product (i.e., r0, r1, r2, and r3 in Figure 1) is located in range (0~0xFFFFFFF). Two pre-computed LUTs within 7681 are constructed. Each LUT receives 8-bit long input. The first input is located within 17-th~24-th bits (i.e., r2 in Figure 1). By passing the LUT, the 8-bit input is transformed into 13-bit wise result (≈$(\left(IR\;div\;2^{16}\right)\;mod\;2^{8})\;\mathrm{mod}\;7681$). The output is added to the intermediate result (i.e., r0 and r1 in Figure 1) and this may generate 17-bit wise intermediate result (i.e., result of Step 2 in Figure 1; addition of 13-bit result and 16-bit result). Afterward, the two separate parts passed to the second LUT come from different variables: The 14-th~17-th bits are from the result after Step 2 in Figure 1, while the 25-th~28-th bits are from the input variable r3 (Two LUTs only require 1 KB ($2^{8}\times 2+2^{8}\times 2$). Both LUTs are stored in the FLASH memory of target 8-bit AVR microcontrollers. Considering that 8-bit AVR platforms support the FLASH memory, which ensures write-only storage. The size of FLASH memory is ranging from 128~384 KB, depending on microcontrollers. The storage for LUTs (1 KB~1.5 KB) is negligible on the target processors with 128~384 KB.). The output of second LUT is 13-bit wise results (≈$(IR\;div\;2^{13})\;mod\;7681$) and this is added to remaining intermediate results (i.e., t1 and t0 in Figure 1). The addition outputs 14-bit wise results. Previous work requires final reduction after two times of LUT accesses, while proposed method terminates the computation after only two times of LUT accesses. By removing the final reduction step, the performance is improved, further.

The detailed modular reduction is given in Figure 1. The intermediate result of product is kept in four registers (r3,r2,r1,r0). Different colors represent different registers, where the register is 8-bit long. The colored block and white block represent used and not used for computation, respectively. The proposed reduction on 7681 is given as follows:

First, LUT access with the variable (r2) is executed. This operation outputs 13-bit wise results (i.e., s1 and s0). Afterward, outputs (i.e., s1 and s0) are added to intermediate results (i.e., r1 and r0). The addition of 16-bit and 13-bit operands outputs the 17-bit wise result. Then, values below 13-bit are extracted from intermediate results (i.e., k2,k1,k0). The 13-bit result is stored in variables (t1,t0). The highest limb (r3) and the other 4-bit wise intermediate result (i.e., (k2,k1)&0x1E0) are combined to generate the 8-bit wise value. Second, LUT access with the generated 8-bit input is performed. This operation generates 13-bit wise results (i.e., s1 and s0). Finally, intermediate results (t1,t0) and LUT outputs (i.e., s1 and s0) are added together. This may generate final 14-bit long results.

In Algorithm 2, the LUT-based modular reduction in source code level is described. Firstly, in Step 1~13, MOV-and-ADD multiplication technique is used to perform the 16-bit wise multiplication. The 28-bit intermediate result is obtained and stored in 4 8-bit registers (R18, R19, R20, R21). Afterward, the LUT-based reduction operation is performed. The LUT input and output are 8-bit long and 16-bit long, respectively.
**Algorithm 2** LUT-based modular reduction in source code (mod 7681)**Input:** operands R22, R23, R24, R2517: LDI R31, hi8(LUT1_H)18: LPM R23, Z**Output:** results {R24, R25}19: ADD R18, R221: CLR R26{MOV-and-ADD}20: ADC R19, R232: MUL R24, R2221: ADC R20, R20{Register re-use}3: MOVW R18, R04: MUL R25, R2322: MOV R30, R195: MOVW R20, R023: ANDI R19, 0X1F24: LSR R206: MOVW R18, R025: ROR R307: ADD R19, R08: MOVW R18, R026: ANDI R30, 0XF09: MOVW R18, R027: ADD R30, R2128: LDI R31, hi8(LUT2_L){LUT access}10: MOVW R18, R029: LPM R24, Z11: MOVW R18, R012: ADC R20, R130: LDI R31, hi8(LUT2_H)13: ADC R21, R2631: LPM R25, Z14: MOV R30, R2032: ADD R24, R1815: LDI R31, hi8(LUT1_L){LUT access}33: ADC R25, R1916: LPM R22, Z34: CLR R1

The Algorithm 1 is fully implemented in assembly language for high speed implementation of NTT. It is possible to implement them by calling each function, independently. Definitely, this approach is efficient for program maintenance. However, this approach has disadvantages, in terms of performance. First, each operation requires function call routine. The function call routine requires stack management and program jump flow. After performing the function, the return process is also required. Second, all variables should be kept in memory space. This process generates additional overheads for memory load and store in the variable access. By using assembly implementation, we reduce the number of function call and memory accesses, significantly.

**Figure 2:**
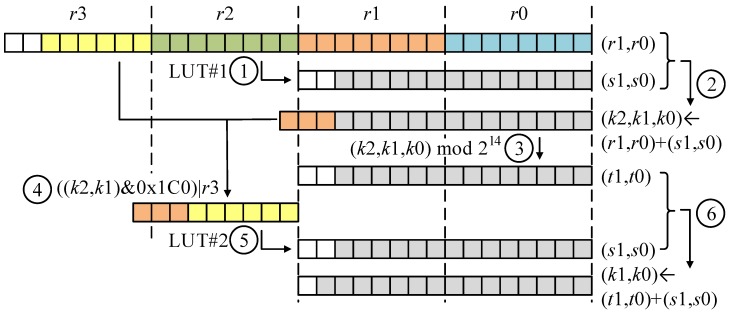
Look-Up Table-based Fast Reduction for q=12,289, ①: LUT access; ②: addition; ③: modulo; ④: concatenation; ⑤: LUT access; ⑥: addition.

When target prime modulus is q=12,289, the operand is located within range (0~0x7FFF). The intermediate result of partial product is located in range (0~0x3FFFFFFF). Two pre-computed LUTs within q=12,289 are constructed. Each LUT receives 8-bit long input. First input is located within 17-th~24-th bits. By passing the LUT, the 8-bit input is transformed into 14-bit wise result (≈$(\left(IR\;div\;2^{16}\right)\;mod\;2^{8})\;\mathrm{mod}\;12,289$). The output is added to the intermediate result (i.e., r0 and r1 in Figure 2) and this may generate 17-bit wise intermediate result (i.e., Step 2 of Figure 2; addition of 14-bit result and 16-bit result). Afterward, the two separate parts passed to the second LUT come from different variables, where 15-th~17-th bits are from the result after Step 2 in the Figure 2, while the 25-th~30-th bits are from the input variable r3 (Both LUTs only require 1.5 KB ($2^{8}\times 2+2^{9}\times 2$) memory space. Two LUTs are stored in the FLASH memory space). The output of second LUT is 14-bit wise results (≈$(IR\;div\;2^{14})\;mod\;12,289$) and this is added to remaining intermediate results (i.e., t1 and t0 in Figure 2). The addition outputs 15-bit wise results.

The detailed modular reduction on 12,289 is given in Figure 2 and the proposed reduction on 12,289 is given as follows:

Firstly, LUT access with the variable (r2) is executed. This operation outputs 14-bit wise results (i.e., s1 and s0). Afterward, outputs (i.e., s1 and s0) are added to intermediate results (i.e., r1 and r0). The addition of 16-bit and 14-bit operands outputs the 17-bit wise result. Then, values below 14-bit are extracted from intermediate results (i.e., k2,k1,k0). The 14-bit result is stored in variables (t1,t0). The highest limb (r3) and the other 3-bit wise intermediate result (i.e., (k2,k1)&0x1C0) are combined to generate the 9-bit wise value. Second, LUT access with the generated 9-bit input is performed. This operation generates 14-bit wise results (i.e., s1 and s0). Finally, intermediate results (t1,t0) and LUT outputs (i.e., s1 and s0) are added together. This may generate final 15-bit long results.

The detailed source code for LUT-based fast modular reduction is given in Algorithm 3.
**Algorithm 3** LUT-based modular reduction in source code (mod 12,289)**Input:** operands R22, R23, R24, R2517: LDI R31, hi8(LUT1_H)18: LPM R23, Z**Output:** results {R24, R25}19: ADD R18, R221: CLR R26{MOV-and-ADD}20: ADC R19, R232: MUL R24, R2221: ADC R20, R20{Register re-use}3: MOVW R18, R04: MUL R25, R2322: MOV R30, R195: MOVW R20, R023: ANDI R19, 0X3F24: ANDI R20, 0X016: MUL R24, R2325: ANDI R30, 0XC07: ADD R19, R08: ADC R20, R126: ADD R30, R219: ADC R21, R2627: LDI R31, hi8(LUT2_L){LUT access}28: ADD R31, R2010: MUL R25, R2229: LPM R24, Z11: ADD R19, R012: ADC R20, R130: LDI R31, hi8(LUT2_H)13: ADC R21, R2631: ADD R31, R2032: LPM R25, Z14: MOV R30, R2015: LDI R31, hi8(LUT1_L){LUT access}33: ADD R24, R1816: LPM R22, Z34: ADC R25, R1935: CLR R1

In Step 1~13, two 15-bit coefficients are multiplied and output 30-bit wise intermediate result. The result is stored in 4 8-bit registers (R18, R19, R20, R21). After the multiplication, the modular reduction is performed. The first LUT receives 8-bit input and generates 16-bit output.

In Step 14~15, bits ranging from 17-th to 24-th (R20) is assigned to the lower 8-bit address (R30). The higher 8-bit address of LUT1_L is assigned to the register (R31). In Step 16, FLASH memory access to first LUT is performed with LPM instruction. The LPM instruction consumes 3 clock cycles per each byte. In Step 17~18, the higher part of LUT1 (i.e., LUT1_H) is loaded. This is separated access to aligned memory address. In Step 19~21, the output of LUT1 and intermediate result are added. The carry bit generated in Step 20 is stored in the register (R20). Thereafter, in Step 22~25, two intermediate results are concatenated. In Step 26~32, LUT2 access is performed in the aligned memory access method. Finally, reduced results and intermediate results are added together. This approach ensures 15-bit wise intermediate results.

In order to accelerate the memory access, we exploited two different optimized memory access techniques. First method is the memory access in an aligned format. The higher 8-bit address is always constant where the offset is 8-bit long. The lower byte is only updated with different offsets to access the memory space. The detailed descriptions are as follows (These methods are defined, where R1, R24, R25, R30, R31, R26, and Z are zero value, first input value, second input value, lower part of memory address, higher part of memory address, result, and Z pointer, respectively).

Initialization of 8-bit aligned access is

MOV R30, R24→LDI R31, hi8(LUT)→LPM R22, Z

The second step of 8-bit aligned access is

MOV R30, R24→LPM R22, Z

**Figure 3:**
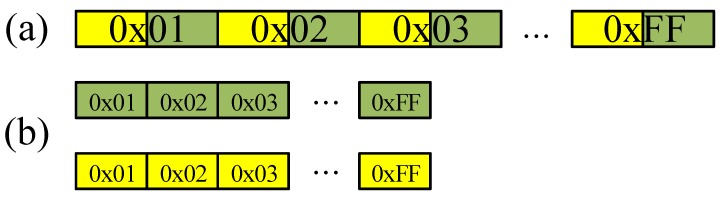
Comparison of LUT construction, (**a**) previous method, (**b**) proposed separated memory access. Yellow and green blocks represent higher and lower parts for LUT, respectively.

The second approach is a separated memory access for 16-bit wise LUT outputs. The LUT for targeted modulus outputs 14-bit and 15-bit wise results for 7681 and 12,289, respectively. The LUT access requires 2-byte aligned offsets to obtain the 14-bit or 15-bit result, which means 9-bit offsets. In this access pattern, the aligned memory access is not feasible since the offset size increases from 8-bit to 9-bit. In order to resolve this issue, we separated the one 16-bit LUT output into two 8-bit parts. The first output is for lower 8-bit result and the second output is for higher 8-bit result. The detailed method is described in Figure 3. Unlike the previous LUT construction, separated two LUTs are constructed. Under this LUT setting, the aligned memory access can be efficiently performed.

The proposed modular reduction method is a generic approach for any primes for lattice-based schemes. For this reason, the proposed method can be extended to other primes without difficulty. Definitely, the proposed method is working for lattice-based NIST PQC candidates, such as NewHope and CRYPSTALS-KYBER [33,34].

#### 3.2.1. Discrete Gaussian Sampling

Discrete Gaussian sampling is an important part of Ring-LWE scheme. For the fast sampling method, we adopted the Knuth–Yao sampler method with byte-scanning [28,35]. This byte-scanning method samples the value in byte-wise rather than bit-wise. However, the original sampling is not a secure approach against timing attack and simple power analysis. The sampler performs the large part with the LUT access. When the proper result is obtained, the sampling is terminated. For this reason, the timing is highly related with the input value (i.e., secret value). In order to resolve this issue, the random shuffling method after random sampling is used [35]. The approach firstly samples whole results. Afterward, whole results are randomly shuffled with random numbers. The random shuffling technique efficiently removes the relation between random samples and timing information. The attack success ratio is reduced from 1 to $\frac{1}{256}$. However, this countermeasure is also vulnerable to sophisticated side channel attack [36]. For this reason, the target application of our approach is limited to simple IoT nodes with basic security level.

#### 3.2.2. AES-Based Pseudo Random Number Generator

The random number generation is highly related with the security of Ring-LWE schemes. Previous Ring-LWE implementations adopted AES-based Pseudo Random Number Generator (PRNG) algorithm (Available in http://www.atmel.com/Images/article_random_number.pdf). The PRNG algorithm runs the AES block cipher in the counter mode and uses the output as random numbers. The recent 8-bit AVR ATxmega128A1 microcontroller features an AES cryptography accelerator that performs AES-128-based data encryption with reasonably fast computation (Computation takes about 375 clock cycles for 128-bit plaintext) and small memory footprint for AES data and control flow management program. The hardware-assisted AES-based counter mode outperforms software-based implementations of the AES block cipher (about 3,521 clock cycles for 128-bit plaintext).

Furthermore, the AES cryptography accelerator and Arithmetic Logic Unit (ALU) of microcontroller can be independently executed in the target machine, which hides the latency for the AES encryption into the arithmetic execution [28]. The detailed hardware AES encryption is as follows. The hardware accelerator firstly sets the key into the 0x00C3 address. The text is loaded to the 0x00C2 memory address. Afterward, the 0x00C0 memory address is set to 0x80 value to perform the AES-128 encryption. This operation only takes 375 clock cycles for 128-bit plaintext. During this period, other operations can be performed simultaneously. The termination of AES encryption is by checking the 0x00C1 memory address. When the value in the memory is below 0x80 value, it indicates that the encryption is completed.

However, the AES accelerator of the ATxmega128A1 can only support 128-bit key, which is not sufficient for long-term security, such as 192-bit and 256-bit security levels. However, previous works utilized the 128-bit AES hardware accelerator for 256-bit scheme in order to achieve high performance by sacrificing the security [27,29,30]. In [28], they only used the software AES from the AVR Crypto Lib for the long-term security level (i.e., 256-bit security level). The AES-256 encryption requires 3521 clock cycles to encrypt a block under a 256-bit key (Available in http://avrcryptolib.das-labor.org/trac). Unlike previous works, we adopted the most recent optimized implementation by [32]. The implementation utilized the unique feature of counter mode (CTR) of AES block cipher. During the counter mode of operation, the small fraction of data is updated and the remaining part is kept without changes. The method generates the cache table first and the cache table is used for skipping Round 0, Round 1, and part of Round 2. For the AES-256 case, the required clock cycles are 3184. This implementation is 9.5% faster than previous work by AVR Crypto Lib.

## 4. Performance Evaluation

This section presents performance results of proposed Ring-LWE implementation on the 8-bit AVR microcontroller. We first describe the experimental platform in Section 4.1. Afterward, we show a comparison result with previous modular multiplication and NTT implementations in Section 4.2. Finally, we show a comparison result with the previous Ring-LWE implementation in Section 4.3.

### 4.1. Experimental Platform

The proposed implementation is evaluated on the ATxmega128A1 microcontroller (i.e., Xplain board). The target microcontroller has a maximum frequency of 32 MHz, 128 KB FLASH program memory, and 8 KB SRAM. The microcontroller supports an 128-bit AES hardware crypto-accelerator. The target platform can be used in a wide range of applications, such as hand-held battery applications, remote controller, as well as some medical devices. The main structure and interface of Ring-LWE scheme are written in C language while the core operations, such as modular arithmetic and AES encryption is implemented in Assembly language. For the LUT-based approach, constant LUT variables are stored in FLASH program memory. Both LUTs require 1.0 KB and 1.5 KB for saving the pre-computed results of 128-bit and 256-bit security levels, respectively. The memory access for FLASH program memory consumes 3 clock cycles for each byte access (i.e., LPM). We complied our implementation with speed optimization option ‘-O3’ on Atmel Studio 7.0.

### 4.2. Comparison of Modular Multiplication and NTT

Table 1 summarizes execution timing (clock cycles) of modular multiplication and NTT for short-term security (i.e., 128-bit) long-term security (i.e., 256-bit) levels. First, various previous implementations, including [25,26,27,28] failed to achieve the constant-time solution. The execution timing is different depending on secret values. With this information, the hacker can perform timing attack or simple power analysis, which extracts some secret values out of them.

The previous work by Liu et al. introduced the secure approach with tiny Montgomery reduction [29]. They perform the Montgomery reduction to reduce the 28/30-bit variables to 14/15-bit results. They achieved 73 and 70 clock cycles for modular multiplication of 128-bit and 256-bit security levels. With these operations, NTT operations for 128-bit security and 256-bit security require 194,145 and 516,971 clock cycles, respectively. The implementation achieved constant timing. However, the complexity of *n*-word Montgomery reduction is generally $n^{2}+n$, which is still high computation overheads on such a low-end devices, such as 8-bit AVR microcontrollers. The modular multiplication can be efficiently optimized to the memory access as proposed in following works. In ICISC’17, Seo et al. suggested novel LUT-based approach to achieve high performance and constant timing of modular multiplication [30]. The algorithm consists of two LUT accesses and one time of final reduction. They achieved 57 and 66 clock cycles for modular multiplication of 128-bit and 256-bit security levels. With these operations, NTT operations for 128-bit security and 256-bit security require 158,607 and 403,224 clock cycles, respectively. The implementation also achieved constant timing. Even though the previous LUT-based implementation achieved significant performance enhancements, we further optimized this approach in this paper.

As shown in the Table 1, the proposed modular multiplication with 7681 and 12,289 primes only requires 46 and 47 clock cycles for 128-bit and 256-bit security level, respectively. These results are 11 and 19 clock cycles smaller than previous approaches [30]. Definitely, the proposed NTT operation also shows higher performance than the previous implementation, significantly. The NTT operation only requires 144,325 and 344,288 cycles for 128-bit and 256-bit security implementations, respectively. Results of NTT for short-term and long-term security is 9.0% and 14.6% faster than previous works [30], respectively. The proposed implementation also achieved the constant timing, which is secure against timing attack and simple power analysis.

### 4.3. Comparison of Ring-LWE

The discrete Gaussian sampler is limited to 12σ to achieve a high precision statistical. These parameter sets were widely used in most of the previous software implementations [24,25,26,28,29,30]. Detailed parameters for target implementations are given in Table 2.

Table 3 compares software implementations of 128-bit and 256-bit security lattice-based cryptosystems on the 8-bit AVR microcontroller, respectively. Previous implementations are fairly compared with our implementations [25,26,27,28,29,30]. The proposed 128-bit security implementation requires 144 K, 524 K, and 659 K cycles for NTT, key generation, and encryption, respectively. NTT implementation and encryption are faster than previous work by 9.0 % and 3.1 %, respectively.

When it comes to 256-bit security implementation with AES software implementation, NTT, key generation, and encryption operations require 344 K, 1775 K, and 2042 K clock cycles, respectively. The NTT operation is faster than the previous work by 14.6%. However, the encryption is 14.1% slower than previous work. This is because the proposed implementation utilized the software-based AES-256 encryption. The previous work uses the hardware-based AES-128 encryption.

For the comparison, the proposed 256-bit security implementation with AES hardware accelerator requires 344 K, 1325 K, and 1430 K clock cycles for NTT, key generation, and encryption, respectively. NTT and encryption are faster than previous works by 14.6% and 18.4%, respectively.

The performance of NTT operation is highly optimized, compared with previous work [30], since the proposed method optimizes the final reduction of modular reduction. With the optimized implementation of NTT and random sampling, the proposed method outperforms the previous work by 3.1% and 18.4% for 128-bit and 256-bit security level, respectively. The proposed method is also secure against simple power analysis and timing attack by ensuring the constant timing. The drawback of proposed method is utilization of memory. For better memory utilization, the memory-efficient implementation should be considered. Furthermore, this approach is only secure against the side channel attack with the architecture without cache memory. When the architecture supports the cache memory, fast reduction or Montgomery reduction without LUT is suitable for the constant implementation.

## 5. Conclusions

This paper presents optimization methods for Ring-LWE implementations. In particular, compact modular multiplication methods for 128-bit (7681) and 256-bit (12,289) security levels are proposed. With modular multiplication methods, NTT operation and its applications (Ring-LWE key generation and encryption) are significantly improved on the low-end 8-bit AVR microcontroller. Furthermore, the optimized software-based AES-256 improved the performance of sampling significantly. The proposed NTT implementation achieved new speed records for secure and robust 128-bit and 256-bit Ring-LWE encryption implementations on target platforms. The future works are optimized implementation on high-end IoT devices, such as ARMv7 Cortex-A and ARMv8 Cortex-A processors. These processors support parallel computation (i.e., SIMD instructions), which efficiently performs multiple data in single instruction. We will also investigate the further optimization of proposed method, in terms of sampling and NTT operations together with side channel attack on low-end 8-bit AVR microcontrollers.

## Figures and Tables

**Table 1:** Execution time of modular multiplication and Number Theoretic Transform (NTT) (in clock cycles), where 128-bit security represents (n:256, q:7681) and 256-bit security represents (n:512, q:12,289) on 8-bit AVR processors, e.g., ATxmega64, ATxmega128.

Implementation	128-bit Security			256-bit Security		
	MOD MUL	NTT	Const	Mod MUL	NTT	Const
Boorghany et al. [26]	N/A	1,216,000	–	N/A	2,207,787	–
Boorghany et al. [25]	N/A	754,668	–	N/A	N/A	–
Pöppelmann et al. [27]	N/A	334,646	–	N/A	855,595	–
Liu et al. [28]	N/A	193,731	–	N/A	441,572	–
Liu et al. [29]	73	194,145	√	70	516,971	√
Seo et al. [30]	57	158,607	√	66	403,224	√
This work	46	144,325	√	47	344,288	√

**Table 2:** Experiment parameters for Ring-LWE implementations.

Security Level	*n*	*q*	σ
128-bit	256	7681	$11.31/\sqrt{2\pi }$
256-bit	512	12,289	$12.18/\sqrt{2\pi }$

**Table 3:** Performance comparison of software implementation of 128-bit and 256-bit security level lattice-based cryptosystems on 8-bit AVR processors, e.g., ATxmega64, ATxmega128.

Implementation	NTT/FFT	Key-Gen	Enc	Secure	PRNG
Implementations of 128-bit security level					
Boorghany et al. [26]	1,216,000	N/A	5,024,000	–	128-bit AES H/W
Boorghany et al. [25]	754,668	N/A	3,042,675	–	56-bit DES S/W
Pöppelmann et al. [27]	334,646	N/A	1,314,977	–	128-bit AES H/W
Liu et al. [28]	193,731	589,900	671,628	–	128-bit AES H/W
Liu et al. [29]	194,145	N/A	796,872	√	128-bit AES H/W
Seo et al. [30]	158,607	N/A	680,796	√	128-bit AES H/W
This work (H/W)	144,325	524,211	659,603	√	128-bit AES H/W
Implementations of 256-bit security level					
Boorghany et al. [25]	2,207,787	N/A	N/A	–	56-bit DES S/W
Pöppelmann et al. [27]	855,595	N/A	3,279,142	–	128-bit AES H/W
Liu et al. [28]	441,572	2,165,239	2,617,459	–	128-bit AES S/W
Liu et al. [29]	516,971	N/A	1,975,806	√	128-bit AES H/W
Seo et al. [30]	403,224	N/A	1,754,064	√	128-bit AES H/W
This work (H/W)	344,288	1,325,171	1,430,601	√	128-bit AES H/W
This work (S/W)	344,288	1,775,475	2,042,474	√	256-bit AES S/W
